# Development of an Error Model for a Factory-Calibrated Continuous Glucose Monitoring Sensor with 10-Day Lifetime

**DOI:** 10.3390/s19235320

**Published:** 2019-12-03

**Authors:** Martina Vettoretti, Cristina Battocchio, Giovanni Sparacino, Andrea Facchinetti

**Affiliations:** Department of Information Engineering, University of Padova, 35131 Padova, Italy; martina.vettoretti@dei.unipd.it (M.V.); cristina.battocchio@studenti.unipd.it (C.B.); gianni@dei.unipd.it (G.S.)

**Keywords:** continuous glucose monitoring, measurement error, calibration error, modeling, glucose sensor, parameter estimation

## Abstract

Factory-calibrated continuous glucose monitoring (FC-CGM) sensors are new devices used in type 1 diabetes (T1D) therapy to measure the glucose concentration almost continuously for 10–14 days without requiring any in vivo calibration. Understanding and modelling CGM errors is important when designing new tools for T1D therapy. Available literature CGM error models are not suitable to describe the FC-CGM sensor error, since their domain of validity is limited to 12-h time windows, i.e., the time between two consecutive in vivo calibrations. The aim of this paper is to develop a model of the error of FC-CGM sensors. The dataset used contains 79 FC-CGM traces collected by the Dexcom G6 sensor. The model is designed to dissect the error into its three main components: effect of plasma-interstitium kinetics, calibration error, and random measurement noise. The main novelties are the model extension to cover the entire sensor lifetime and the use of a new single-step identification procedure. The final error model, which combines a first-order linear dynamic model to describe plasma-interstitium kinetics, a second-order polynomial model to describe calibration error, and an autoregressive model to describe measurement noise, proved to be suitable to describe FC-CGM sensor errors, in particular improving the estimation of the physiological time-delay.

## 1. Introduction

Type 1 diabetes (T1D) is an autoimmune disease in which the pancreas loses the capability of producing insulin, the hormone stimulating the absorption of blood glucose (BG) by the body tissues. The lack of insulin production results in persistently high BG concentration that needs to be treated by exogenous insulin administrations. In order to tune their exogenous insulin doses, people with diabetes need to frequently monitor their BG concentration, especially to avoid too high BG concentrations, which in the long term can drive to microangiopathy or macrovascular disease, and too low BG concentrations, which in the short term can cause seizure, coma or even death. 

The conventional BG meters are the so-called self-monitoring of blood glucose (SMBG) devices, i.e., portable devices by which the patients can measure the glucose concentration in a small drop of capillary blood collected by fingerstick [1]. The main limitation of SMBG systems is that the patients can only collect sparse measurements (typically 3–5 per day), thus missing information on the BG dynamics, and with asymptomatic, but potentially dangerous, high/low BG events remaining undetected. This limitation can be overcome by continuous glucose monitoring (CGM) sensors that allow measuring interstitial glucose (IG) concentration almost continuously (e.g., every 1–5 min) for several consecutive days or weeks [2,3,4,5]. Most popular CGM sensors are the minimally-invasive electrochemical sensors that consist of a needle electrode placed in the subcutaneous tissue of the abdomen or the arm that measures a current signal originated by glucose oxidation, which is then converted to a glucose concentration profile using a conversion function. The glucose readings of the sensor are finally transmitted to a receiver that displays the measurements in real-time to the patient. 

Up until a few years ago, all the CGM sensors needed to be calibrated in vivo, i.e., the patient was required to collect a SMBG value to be inserted in the receiver, where an automated calibration procedure was run to update the parameters of the conversion function [6]. CGM sensors required to perform the calibration procedure periodically, usually every 12 h, to compensate for the time-variability of the sensor’s sensitivity and the biological environment in which the sensor is inserted (factors not accounted into the conversion function). In the last few years, with the improvement of CGM sensing technologies, the calibration requirements of CGM sensors were reduced [7], until the release of factory-calibrated sensors, i.e., CGM sensors that are calibrated only once during the manufacturing process and do not require any in vivo calibration [8,9,10]. The FreeStyle Libre (Abbott Diabetes Care Inc., Alameda, CA, USA) [11,12], launched in 2014, and the Dexcom G6 (Dexcom Inc., San Diego, CA, USA) [13,14], released in 2018, are the two examples of factory-calibrated glucose sensors, although the FreeStyle Libre provides glucose measurements only upon scanning the sensor with the receiver and, thus, it is considered a flash glucose monitoring sensor rather than a proper CGM sensor.

When compared to BG measurements collected very frequently by high-accuracy and precision instruments, CGM sensors are unavoidably affected by error. Several factors were found to affect the accuracy of CGM sensors, such as imperfect calibration, which can result from alterations of the sensor sensitivity because of the foreign body response of the patient’s immune system [15], artefacts because of the compression of the sensor site [16], and interfering substances [17,18] (although the Dexcom G6 was recently shown to be resistant to acetaminophen interference [19]). Another issue concerns the site of measurement of CGM sensors, i.e., the interstitial fluid. Indeed, the BG-to-IG kinetics gives rise to a physiological delay of measurements performed in the IG site, like CGM measurements, compared to BG measurements, because of the glucose diffusion time between the plasma and the interstitium compartments [20]. 

Mathematical models of CGM error are useful tools to quantify and understand the sensor error, and design solutions for enhancing its performance. In addition, models of CGM error can be used to generate reliable synthetic CGM data and perform in silico clinical trials [21] to test in simulation the efficacy of CGM-based therapies and applications [22], like decision-support systems [23,24], bolus calculators [25,26,27], basal insulin modulation algorithms [28,29,30], and artificial pancreas systems [31,32,33]. Some approaches to model the CGM sensor error were proposed in the literature. Breton and Kovatchev [34], for instance, proposed a model for the FreeStyle Navigator (Abbott Diabetes Care, Alameda, CA), in which the error because of BG-to-IG kinetics was described by a first-order linear dynamic model with fixed time-constant, and the measurement noise was described by a first-order autoregressive (AR) model. A second model was proposed by Lunn et al. [35] for the Guardian RT sensor (Medtronic, Northbridge, CA, USA), in which the time-constant of the model describing BG-to-IG kinetics was individualized, and a simple model of the calibration error based on linear regression was included. Another approach was developed by Facchinetti et al. [36] in which, at difference of previous methods, the time variability of sensor calibration error is coped with by the use of a time-variant function. This method was first designed using data collected by Dexcom SEVEN Plus (Dexcom, Inc., San Diego, CA, USA) sensor [36], and then successfully applied to other datasets collected by Dexcom G4 PLATINUM and G4AP [37], Dexcom G5 Mobile [22], and Medtronic Paradigm Veo Enlite [38]. 

All these literature models were developed to describe the error of CGM sensors that required in vivo calibrations, and thus these models were identified in short time windows between consecutive calibrations (e.g., 12 h for Dexcom G4/G5 sensors), considering the error in two different between-calibrations time windows as independent on each other. The direct application of these models to factory-calibrated CGM sensors is not possible, because, as the in vivo calibrations are absent, the error observed in any short time window cannot be considered as independent from the error observed in the rest of the monitoring period. A simple extension of the literature models to the entire lifetime of factory-calibrated sensors is also nonviable. Indeed, the description of the calibration error adopted by literature models has a domain of validity limited to short between-calibrations time windows (typically 12 h), and it could be not suitable to describe the calibration error of a factory-calibrated sensor over the entire sensor lifetime (e.g., 10–14 days). 

Therefore, our aim is to develop a new model to describe the error of the factory-calibrated CGM sensors with domain of validity that covers the entire sensor lifetime. For this purpose, a preliminary model was developed in Vettoretti et al. [39] by applying the methodology of Facchinetti et al. [36] to a small dataset collected in 11 subjects monitored with the Dexcom G6 sensor that measures IG concentration every 5 min for 10 days. In this paper, we derive a new model by exploiting a larger dataset containing 79 Dexcom G6 traces and by applying a methodology similar to the one proposed by Facchinetti et al. [36], with some differences in the modelling framework later described in Section 2, especially the extension of the domain of validity of the model from 12 h to 10 days in order to cover the entire sensor lifetime. Moreover, to overcome the limitations of the two-step identification procedure used in the previous works of Facchinetti et al. [36], which can occasionally lead to suboptimal estimation of some model parameters, in this work we propose a new single-step identification method, in which all the model parameters are estimated simultaneously. Results of the new single-step identification are compared to those of the two-step identification used in the literature in Section 3.

## 2. Materials and Methods

### 2.1. Dataset

**Figure 1:**
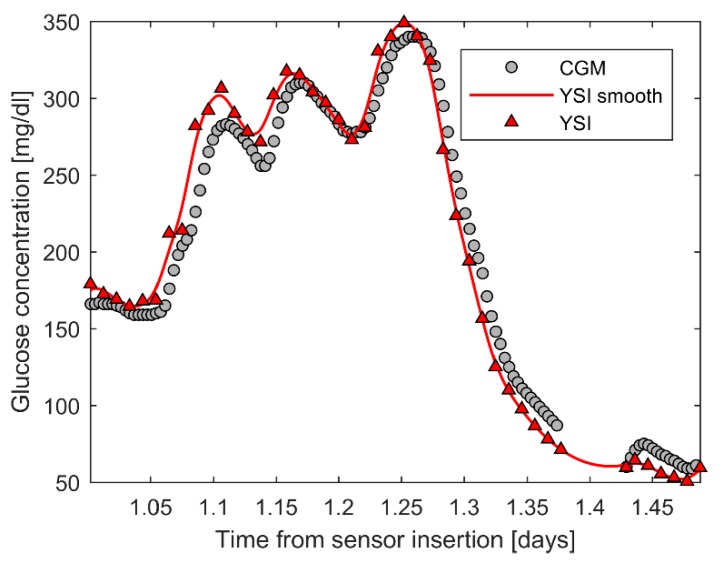
Data preprocessing in a representative subject’s day. Continuous glucose monitoring (CGM) and Yellow Springs Instrument (YSI) measurements are reported by grey circles and red triangles, respectively. The reconstructed YSI smooth profile is reported by the red line.

The original dataset, courtesy of Dexcom Inc., included 177 CGM traces collected in 141 T1D adults by the Dexcom G6 sensor (36 subjects wore two sensors in parallel). The CGM traces, with duration of 10 days, were obtained with factory calibration, i.e., the patients did not perform any in-vivo calibration using SMBG. During the period of CGM monitoring, subjects attended three 12-h clinical sessions in which meal and insulin doses were manipulated to induce large glycemic excursions and reference BG measurements were collected every 15 min by high-accuracy and precision laboratory instrumentation (YSI, Yellow Springs Instrument, Yellow Springs, OH). Clinical sessions were performed at the beginning (either day 1 or 2), in the middle (day 4) and at the end (either day 7 or 10) of CGM sensor lifetime. An example of data collected during an YSI session in a representative subject is shown in Figure 1 (CGM measurements reported by grey circles, YSI measurements figured by red triangles).

### 2.2. Preprocessing

Five sensors were excluded because insufficient data were collected (less than 12 h). Since our purpose is to model the sensor measurement error over the entire monitoring period of 10 days, we also excluded from our analysis all those traces where the third in-clinic session was performed in day 7 rather than in day 10. Indeed, if we developed an error model using such data, the model validity would be restricted to only 7 days. Two more sensors were excluded because of having less than 8 YSI samples collected in day 10. As a result, 79 sensor traces were selected, 18 having YSI sessions in day 1, 4, and 10, and 61 having YSI sessions in days 2, 4, and 10. 

CGM measurements that saturated to the maximum (400 mg/dL) or the minimum (40 mg/dL) value displayed by the Dexcom G6 system were removed. YSI measurements (red triangles in Figure 1) were first analyzed by visual inspection, in order to remove the possible outliers, and then smoothed by the nonparametric Bayesian smoothing algorithm. This smoothing step is necessary to obtain a BG value sufficiently close in time to each CGM measurement (maximum temporal distance of 30 s) to be used as reference for the assessment of CGM error. Briefly, the smoothing algorithm implements the widely known Tikhonov’s regularization [40]: A smooth BG signal (red line in Figure 1) is obtained, on 1-minute evenly-spaced grid, by minimizing a two-term cost function, in which the first term penalizes the distance of the unknown BG signal from the YSI data (measured on a rare/nonuniform sampling grid), while the second one penalizes its variability (given by the sum of squared second differences). A scalar smoothing parameter determines the relative weight of these two terms: The higher this parameter, the smoother the reconstructed BG signal, at the cost of increased bias and larger residuals. As proved by De Nicolao et al. [41], to which we refer the reader for more details on the entire procedure, when embedded in a Bayesian framework this approach allows optimization of the bias-variance trade-off by determining the numerical value of the smoothing parameter via a maximum likelihood criterion. Notably, this allows compensating the unavoidable presence of measurement error on YSI samples (assumed to be white noise with zero-mean and 2% coefficient of variation), while limiting the risk of introducing distortion/bias in the reconstructed BG profile. Nevertheless, when two consecutive YSI measurements were more than 20 min far from each other (gap in the YSI time series), the CGM measurements collected between the two YSI measurements were excluded, as the reconstructed BG profile in such interval was not reliable.

### 2.3. Proposed CGM Error Model

**Figure 2:**
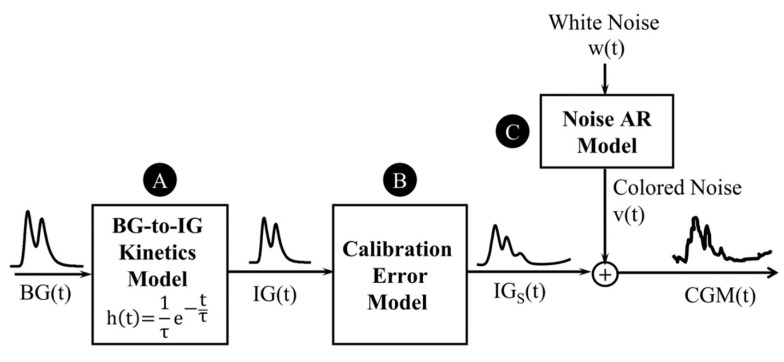
Schematic representation of the modelling framework for factory-calibrated sensors. The final model of CGM measurements, starting from blood glucose (BG) concentration, includes three main components: a model of the effect of BG-to-interstitial glucose (IG) kinetics (block A), the calibration error model (block B) and the random measurement noise model (block C).

The proposed CGM error model, schematized in Figure 2, takes into account the three main error components corresponding to three main sources of error: the effect of BG-to-IG kinetics (block A in Figure 2), the calibration error (block B in Figure 2), and the random measurement noise (block C in Figure 2). 

The proposed model reflects the general structure of the one developed by Facchinetti et al. [36] for CGM sensors requiring periodic in vivo calibrations with two main differences. First, the calibration error model (block B in Figure 2) has been extended to describe the trend of the calibration error over the entire sensor lifetime (10 days), while the validity of the previous model by Facchinetti et al. [36] was limited to short 12-h time windows between two consecutive calibrations. Second, the model was adapted to be identified on a single CGM trace per subject, as in our dataset most of the subjects wore a single CGM sensor. In this regard, the parameters of the BG-to-IG kinetics model (block A, in Figure 2) are considered specific of the subject-sensor couple, rather than subject-specific as in previous works [36,37,38,39]. Moreover, the random measurement noise (block C in Figure 2) is considered as a single noise component, as the splitting of noise into sensor-specific component and common component, performed in previous works [36,37,38,39], necessarily requires multiple parallel sensors per subject. Note that, besides the practical reason mentioned above, this choice was also motivated by the fact that the strong assumption of previous models [36,37,38,39] that the BG-to-IG time-constant is independent of the sensor and the sensor insertion site may not be valid. The three following sections describe in details the models of the three main error sources that compose the proposed CGM error model.

#### 2.3.1. BG-to-IG Kinetics Model

According to previous literature works [42,43], the plasma-interstitium kinetics can be described by a two-compartment model that, assuming the balance between BG and IG in steady-state conditions, is equivalent to the first-order linear dynamic system with impulse response:

(1) h(t)=1τ e−tτ

and diffusion time constant *τ*. Then, the relationship between BG and IG is the following:

(2) BG(t)=h(t)⊗IG(t)

as mentioned above, the time constant *τ*, which is the only parameter of the BG-to-IG kinetics model, is considered as a parameter specific of the subject-sensor coupling.

#### 2.3.2. Calibration Error Model

Systematic over/underestimations because of the factory-calibration error are modelled by the following equation:

(3) $$IG_{s}\left(t\right)=a\left(t\right)\cdot IG\left(t\right)+b\left(t\right)$$

where *IG_s_*(*t*) is the signal virtually measured by the sensor in absence of the random noise and *t* represents the time from sensor insertion. The time-variability of the calibration error over sensor lifetime (10 days) is taken into account by two functions of time, *a*(*t*) and *b*(*t*), for which different candidate models are considered. First, for both *a*(*t*) and *b*(*t*), we consider the polynomial models of order *m* = 0,1,2,3 already used in previous studies [36,37,38,39]:

(4) $$poly_{m}\left(t\right)=\overset{m}{\underset{k=0}{\sum }}p_{k}t^{k}$$

with parameters *p_k_*, *k* = 1,…,*m*. In addition, an exponential function, *exp*(*t*), is considered with three parameters, *p*_0_, *p*_1_, and *p*_2_, representing the initial value, the final value, and the time constant of the exponential. Overall, 25 different candidate calibration error models are considered.

#### 2.3.3. Random Measurement Noise

Finally, we assume that the output signal of the sensor, *CGM*(*t*), is affected by additive measurement noise *v*(*t*):

(5) $$CGM\left(t\right)=IG_{s}\left(t\right)+v\left(t\right)$$

as demonstrated by previous studies [34,35,36], the sensor noise presents a certain level of autocorrelation. Therefore, we consider as candidate models for *v*(*t*) the AR models of order *q* = 1, 2,…, 10:

(6) $$v\left(t\right)=\sum_{k=1}^{q}\alpha_{k}v\left(t-kT\right)+w\left(t\right)$$

where *T* is the CGM sampling period, while the coefficients *α_k_*, *k* = 1,…, *q* and the variance *σ*^2^ of the zero-mean white noise *w*(*t*) guiding the AR process are the unknown model parameters.

### 2.4. Estimation of Model Parameters

As for the model of Facchinetti et al. [36], the model parameters can be estimated in two steps: The first step focused on the identification of the models of BG-to-IG kinetics and calibration error, and the second step for the identification of the noise model. Details on this two-step identification procedure are reported in Section 2.4.1. Furthermore, in this paper we test a new single-step identification strategy, described in Section 2.4.2, in which all the model parameters are estimated simultaneously.

#### 2.4.1. Two-Step Model Identification Procedure

In the first step, the BG-to-IG kinetics model and the calibration error model are identified. In particular, for each candidate calibration error model, $M_{i}=\left(a_{i}\left(t\right),\;b_{i}\left(t\right)\right)$, where $a_{i}\left(t\right)$ and $b_{i}\left(t\right)$ can be either the polynomial or the exponential function, the parameters of $M_{i}$ (*p_k_, k* = 1,…,*N_p_*) and the time constant *τ* are estimated by non-linear least squares, i.e., solving the following minimization problem:

(7) $$\hat{\theta }=\underset{\theta }{\mathrm{argmin}}\left(\sum_{j=1}^{n}{r_{j}}^{2}\right)=\underset{\theta }{\mathrm{argmin}}\left(\sum_{j=1}^{n}{\left(ys_{j}-y_{j}\left(\theta \right)\right)}^{2}\right)$$

where ***θ*** = {*τ*, *p*_0_, *p*_1_,…, *p_Np_*} is the set of model parameters, $r_{j}$ is the residuals of the model (i.e., samples of *v*(*t*)) obtained as difference of the CGM measurements $ys_{j}$ (i.e., samples of CGM(t)) and the model predictions $y_{j}$ (i.e., samples of $IG_{s}\left(t\right)$). The optimal model for *a*(*t*) and *b*(*t*) is selected by minimizing the Bayesian Information Criterion (BIC) index:

(8) $$BIC\left(M_{i}\right)=n\cdot \ln\left(\frac{RSS}{n}\right)+\left(N_{p}+1\right)\cdot \ln\left(n\right)$$

where the first addendum, depending on the residual sum of squares *RSS*, takes into account the goodness of fit, while the second addendum accounts for model complexity. Since Equation (8) holds under the assumption that the residuals are independent and identically normally distributed, a whitening filter is applied to the residuals $r_{j}$ *j* = 1,…,*n* before calculating the *RSS*. Specifically, the transfer function of the whitening filter is the inverse of the transfer function of an AR model of order 2 fitted on the residuals. Indeed, according to previous studies the noise of CGM sensors can be well described by a second-order AR model [36,37,38,39]. By using Equation (8), a BIC value is calculated for each sensor and each model. Then, the optimal calibration error model is chosen as the model allowing the lowest distribution of BIC values.

The second identification step consists of selecting the optimal random measurement noise model and estimating its parameters from the residuals of the first identification step, $r_{j}$ with *j* = 1,…,*n*. Each of the candidate models, i.e., the AR models of order *q* = 1,2,…,10 and coefficients *α_k_* with *k* = 1,…,*q*, is fitted to the residuals by forward-backward least squares. Also in this case, the optimal AR model is selected according to the BIC index. Note that while in some previous works [22,37,39], a different AR model was fitted in each day of CGM monitoring, here a single AR model is fitted for all the monitoring period.

The two-step identification, adopted also by previous studies, facilitates both the choice of the optimal models, which are selected separately, and the search for optimal parameters, as a subset of the total model parameters is estimated in each least-squares problem. Nevertheless, a possible limitation of this approach is that occasionally the estimation of the model parameters with the two-step identification procedure can lead to suboptimal estimates of some parameters, e.g., the diffusion time constant *τ* of the BG-to-IG kinetics model. For this reason in this paper we also propose a single-step identification procedure described in the following section.

#### 2.4.2. Single-Step Model Identification Procedure

In the single-step identification procedure, all the parameters of the model are estimated simultaneously. For simplicity, we consider only the optimal calibration error and measurement noise models selected in the two-step identification. Therefore, the parameter vectors ***θ*** = {*τ*, *p*_0_, *p*_1_,…, *p_Np_*} and ***α*** = {*α*_1_, *α*_2_,…, *α_q_*} are estimated by solving the following least-squares problem:

(9) $$\left[\hat{\theta },\;\hat{\alpha }\right]=\underset{\theta ,\alpha }{\mathrm{argmin}}\left(\overset{n}{\underset{j=1}{\sum }}w{r_{j}}^{2}\right)=\underset{\theta ,\alpha }{\mathrm{argmin}}\left\{\overset{n}{\underset{j=1}{\sum }}{\left[ys_{j}-y_{j}\left(\theta \right)-\overset{q}{\underset{k=1}{\sum }}\alpha_{k}\left(ys_{j-k}-y_{j-k}\left(\theta \right)\right)\right]}^{2}\right\},$$

subject to the stability constraint of the AR model, i.e., the roots of the following characteristic polynomial $A\left(z\right)=1-\alpha_{1}z-\alpha_{2}z^{2}-\ldots -\alpha_{q}z^{q}$ must lie within the unit circle. Note that in the single-step identification procedure a single objective function is minimized, i.e., the sum of squares of the residuals *wr_j_* that are expected to be samples of white noise, as the autocorrelation of sensor noise is already accounted by the AR component of the model. An estimate of the standard deviation of *w*(*t*), i.e., the white noise in input to block C in Figure 2, is obtained a posteriori by calculating the sample standard deviation of the residuals *wr_j_*, *j* = 1,…,*n*.

#### 2.4.3. Implementation Details

Model identification is implemented in Matlab R2017a. In the two-step identification procedure, the estimation of ***θ*** is performed using the function *lsqnonlin* with Levenberg–Marquardt algorithm and initial values for the parameters such that *τ* = 7 min, *a*(*t*) = 1, and *b*(*t*) = 0. The identification of the AR model in the second step is performed using the function *ar* with forward–backward approach. In the single-step identification procedure, ***θ*** and ***α*** are estimated using the function *fmincon* with interior-point algorithm and initial values for the parameters such that *τ* = 7 min, *a*(*t*) = 1, and *b*(*t*) = 0 and the *α_k_* coefficients represent the average model obtained by the two-step identification procedure.

## 3. Results

### 3.1. Model Selection by the Two-Step Identification Procedure

**Figure 3:**
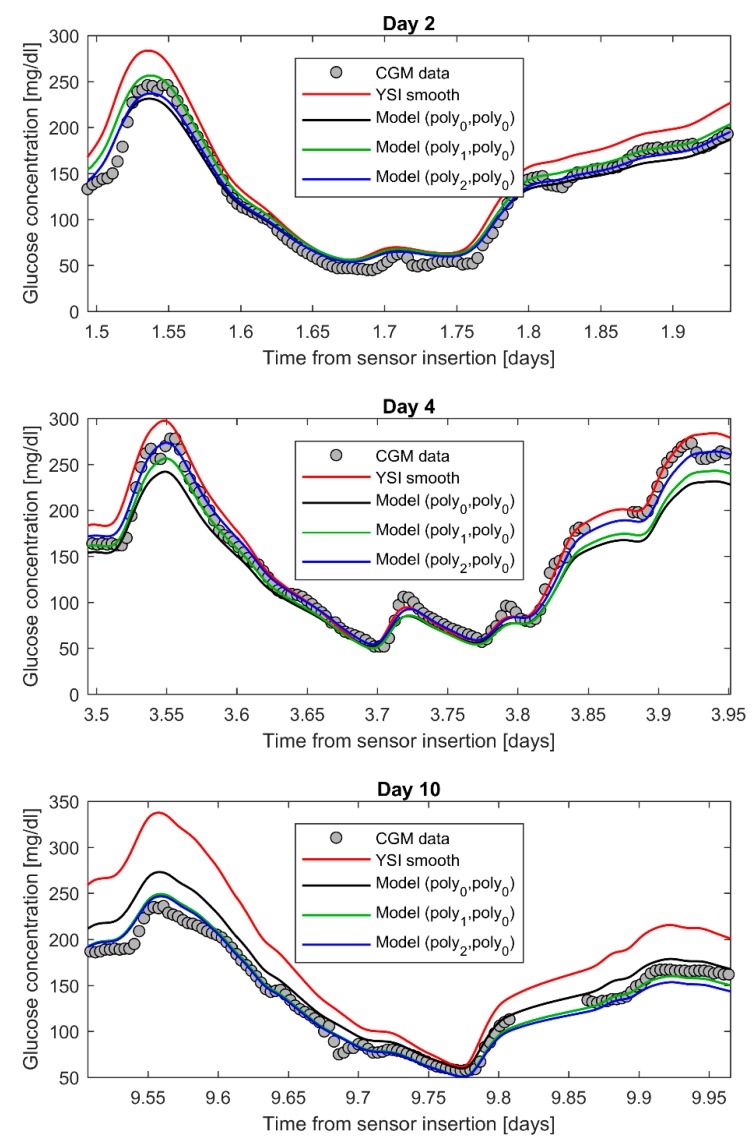
First step of the two-step identification procedure: data fit for a representative subject in days 2 (top panel), 4 (middle panel), and 10 (bottom panel) of CGM monitoring. CGM measurements are reported by grey circles, the YSI smooth profile is the red line, and the CGM profiles predicted by the models (*poly*_0_, *poly*_0_), (*poly*_1_, *poly*_0_), and (*poly*_2_, *poly*_0_) are reported by black, green, and blue line, respectively.

In the first step of model identification, each of the 25 candidate calibration error models (*a*(*t*), *b*(*t*)) is fitted to the data of the 79 sensor traces, using the YSI smooth profile as forcing input (the 25 candidate models are obtained considering both for *a*(*t*) and *b*(*t*) one of the following functions: *poly*_0_, *poly*_1_, *poly*_2_, *poly*_3_, *exp*). In Figure 3, we show an example of data fit obtained with three different models, i.e., (*poly*_0_, *poly*_0_), (*poly*_1_, *poly*_0_), and (*poly*_2_, *poly*_0_), for a representative sensor in day 2 (top panel), 4 (middle panel) and 10 (bottom panel) of CGM monitoring. In this particular example, we can see how the model (*poly*_2_, *poly*_0_) (blue line) allows a better fit of the CGM data (grey circles) compared to the simpler models (*poly*_1_, *poly*_0_) (green line) and (*poly*_0_, *poly*_0_) (black line).

**Figure 4:**
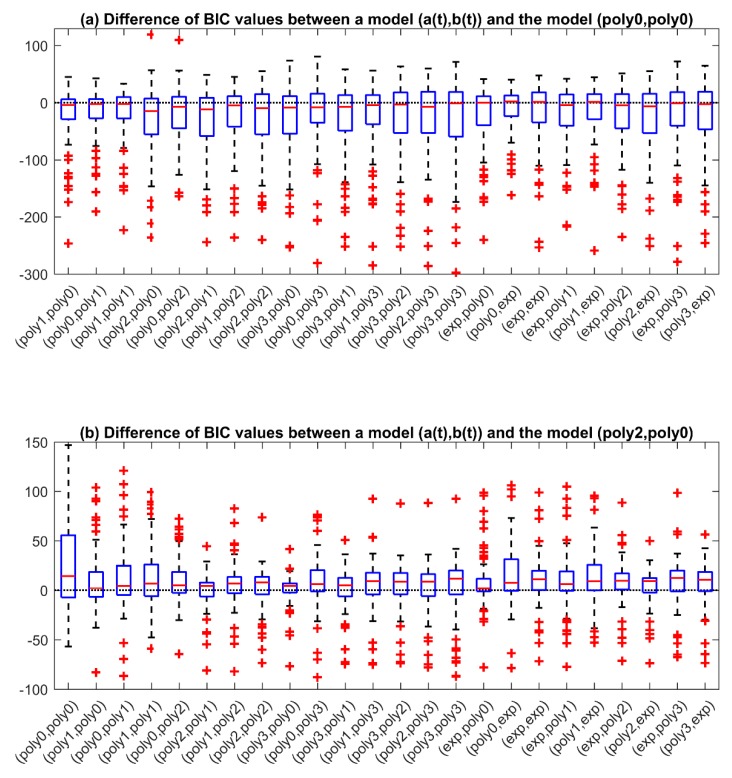
First step of the two-step identification procedure. (**a**) Each boxplot represents the distribution of the difference between the Bayesian Information Criterion (BIC) values obtained with a particular model (*a*(*t*), *b*(*t*)) and the one obtained with the model (*poly*_0_, *poly*_0_). (**b**) Each boxplot represents the distribution of the difference between the BIC values obtained with a particular model (*a*(*t*), *b*(*t*)) and the one obtained with the model (*poly*_2_, *poly*_0_). A negative value means an improvement with respect to the reference model.

After identifying each model, the candidate models are compared in terms of BIC values. In particular, first, we calculated the difference of the BIC values obtained with each time-variant model, (*a*(*t*), *b*(*t*)), and the BIC values obtained with the time-invariant model, (*poly*_0_, *poly*_0_), in which both *a*(*t*) and *b*(*t*) are constant. The distributions of such differences are shown in Figure 4a via boxplot representation. As all the boxplots present a longer negative tail, all the candidate time-variant models perform better than the time-invariant model, suggesting that a temporal component in the model is needed to well describe the time-variability of the calibration error. Since a lower BIC value indicates a better trade-off between data fit and model complexity, we are interested in identifying the model that allows reducing the BIC value most, i.e., the model for which the difference of BIC values is more negative. From the boxplots of Figure 4a, we can see that the greatest BIC reduction is obtained for the model (*poly*_2_, *poly*_0_) and the model (*poly*_2_, *poly*_1_). Between these two models, we select the most simple one, i.e., the model (*poly*_2_, *poly*_0_), and calculate the differences between the BIC values obtained with all the other models and the ones obtained with the model (*poly*_2_, *poly*_0_). The boxplots of such differences, plotted in Figure 4b, show that the model (*poly*_2_, *poly*_0_) outperforms all the other candidate models, because the differences in BIC values are mostly positive for all the models, thus the other candidate models drives to higher BIC values compared to the model (*poly*_2_, *poly*_0_). Based on these results, the optimal calibration error model selected at the end of the first identification step is the model (*poly*_2_, *poly*_0_) whose corresponding equation is:

(10) IGs(t)=(a0+a1t+a2t2)(BG(t)⊗1τe−tτ)+b0.

**Figure 5:**
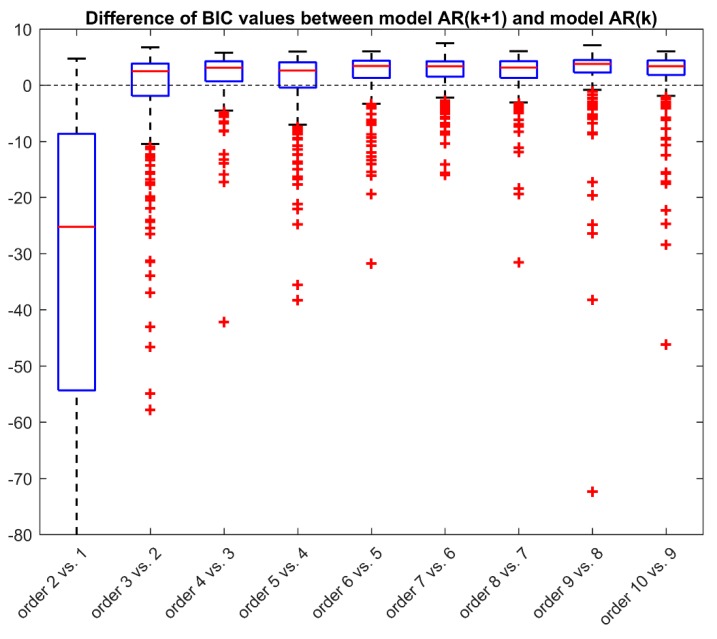
Second step of the two-step identification procedure: boxplots of delta BIC values by increasing the order of the AR model of one unit. The delta BIC values are obtained as the difference between the BIC values obtained with order *k* + 1 and the ones obtained with order *k*, for *k* going from 1 to 9. A negative value means an improvement in the order *k* + 1 compared to the model of order *k*.

In the second step of model identification, the residuals obtained from the first step with the selected optimal model are fitted with AR models of order 1 to 10 in order to identify the best model to describe the sensor residual noise. Also in this case, the different candidate models, AR(*k*) *k* = 1,…,10, are compared in terms of BIC. In Figure 5, we show the boxplot of the difference between BIC values obtained with model AR(*k*+1) and the one obtained with model AR(*k*) for *k* = 1,…,9. When the boxplot of such difference is mostly negative, then increasing the order of the AR model of one unit is advantageous, i.e., the order *k* + 1 is preferable to order *k*; on the contrary, if the difference in BIC values is mostly positive, the increase of the order does not sufficiently improve the data fit, hence the order *k* is preferable to order *k* + 1. As visible in Figure 5, the increase of the order from 1 to 2 allows a great improvement of the performance with a decrease in the BIC values in the majority of the sensors. However, a further increase of the order does not further improve the performance of the model. Indeed, the BIC difference between AR (3) and AR (2) is positive for most sensors, meaning that the order 2 is preferable to order 3. The same happens when further increasing the order. Therefore, these results suggest that the optimal model for describing the sensor residual noise, *v*(*t*), is the AR model of order 2 whose equation is the following:

(11) $$v\left(t\right)=\alpha_{1}v\left(t-T\right)+\alpha_{2}v\left(t-2T\right)+w\left(t\right)$$

where *w*(*t*) is the zero-mean white noise with standard deviation *σ* and *T* is the CGM sampling time, in case of the Dexcom G6 *T* = 5 min.

### 3.2. Two-Step vs. Single-Step Model Identification Procedure

After selecting the optimal calibration error and noise models, we repeated the identification of the optimal complete model using the single-step identification procedure and then we compared the results with those of the two-step identification procedure. 

**Figure 6:**
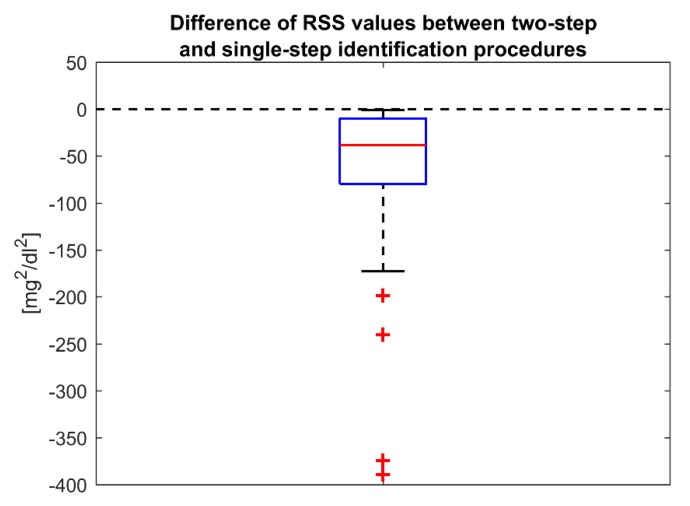
Two-step vs. single-step identification procedure of the optimal model (*a*(*t*) = *poly*_2_, *b*(*t*) = *poly*_0_, *v*(*t*) = AR(2)). Boxplot representation of the difference of between the *RSS* values obtained with the single-step identification and the ones obtained with the two-step identification procedure. A negative value means an improvement of data fit with the single-step identification procedure.

First, we compared the two identification strategies in terms of data fit. By analyzing the root mean square error (RMSE) between the CGM measurements and model predictions, we found that both identification procedures well fit the data, without remarkable differences between the two methods (mean [minimum, maximum] RMSE equal to 3.27 [1.41, 6.69] mg/dL for the two-step identification procedure vs. 3.23 [1.40, 6.56] mg/dL for the single-step identification procedure). However, the boxplot of Figure 6, which represents the difference between the *RSS* obtained with the single-step and the one obtained with two-step identification procedure, evidences that for all the sensors the single-step identification procedure reduces the *RSS*, i.e., improves the data fit. Although no dramatic differences were found between the two *RSS* distributions, the median reduction of *RSS* obtained with the single-step identification procedure is statistically significant according to the signed test (*p*-value < 0.0001). 

**Figure 7:**
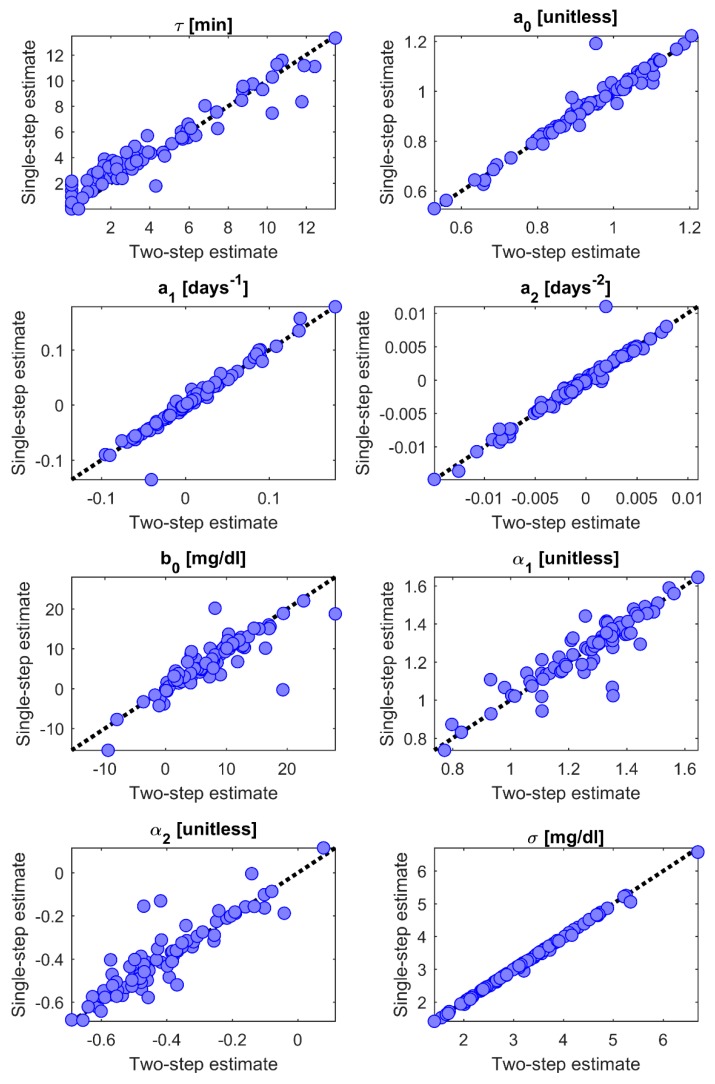
Two-step vs. single-step identification procedure of the optimal model (*a*(*t*) = *poly*_2_, *b*(*t*) = *poly*_0_, *v*(*t*) = AR(2)). Comparison between the parameters’ estimates obtained with the two-step identification procedure (*x*-axis) and the ones obtained with the single-step identification procedure (*y*-axis).

In the scatterplots of Figure 7, we can check the agreement between the parameters’ estimates obtained with the two-step identification procedure (x-axis) and those obtained with the single-step identification procedure (y-axis). A very good agreement between the two estimates is obtained for *a*_0_, *a*_1_, *a*_2_, and *σ*, with a correlation coefficient *ρ* > 0.97 and the scatterplot on top of the diagonal line. For *b*_0_, α_1_, and α_2_, the plot results more scattered around the diagonal line, and the correlation coefficient is still high but lower than for the other parameters (*ρ* = 0.86–0.91). As far as the time-constant *τ* is concerned, the correlation between the two estimates is high (*ρ* = 0.97), but looking at the scatterplot of Figure 7 we can note that the single-step identification procedure tends to estimate higher values of *τ* compared to the two-step identification procedure. In particular, while with the two-step identification procedure there is a considerable number of sensors for which the *τ* estimate is close to 0 (14 sensors with *τ* < 1 min), in the single-step identification procedure this number is significantly reduced (7 sensors with *τ* < 1 min).

In Table 1, for each model parameter we report the median [interquartile range] of the estimates returned by the two identification methods, as well as the percentage of sensors for which the coefficient of variation (CV) of the estimation error was lower than 10% and 30%. The results in Table 1 confirm that the estimates of *τ* obtained with the single-step identification procedure are slightly higher than those of the two-step identification procedure, with a particular increase of the 25th percentile from 1.66 min to 2.39 min. For all the other parameters, the median and the interquartile range of the estimates are similar with the two identification methods. Note that while the median value of the *a*_2_ parameter is very close to zero, this parameter actually assumes a large range of values for different sensors, as shown in the corresponding panel of Figure 7. 

Overall, the precision of the parameters’ estimates is satisfactory for both the two-step and the single-step identification procedure, with some differences between the two methods. The single-step identification increases from 87% to 94% the number of sensors with CV < 30% for *τ*, and from 30% to 95% the number of sensors with CV < 10% for α_2_. However, the precision of the estimates for *a*_1_, *a*_2_, and *b*_0_ is lower with the single-step identification procedure compared to the two-step identification procedure. Such a deterioration can be due to the fact that a greater number of parameters are estimated simultaneously in the single-step identification procedure.

## 4. Discussion

In this work, we developed a new model to describe the error of a factory-calibrated CGM sensor, the Dexcom G6, by using a methodology similar to the one of Facchinetti et al. [36], but with some important differences. First, we dropped the assumption that the time constant of the BG-to-IG kinetics is a subject-specific parameter, and we considered it as a parameter specific of the particular subject-sensor coupling. The implication of this assumption is that each sensor is considered independently of the others in the identification process, thus a different value of *τ* is estimated for each sensor and a single sensor-specific noise component is identified. Second, we extended the model domain of validity to the entire sensor lifetime of 10 days. In this regard, we considered 25 different calibration error models to describe the time-variability of the calibration error over the sensor lifetime, including not only the polynomial models tested in the literature but also a new exponential model. Finally, we also tested a new single-step identification method in order to overcome the possible limitations of the two-step identification approach used previously.

As far as the identification of the BG-to-IG kinetics is concerned, in this study we identified a median *τ* value of about 3.8 min. This value is considerably lower than the one estimated in the multitracer and microdialysis experiment of Schiavon et al. [43], which was around 11 min. The *τ* value estimated in our work is also lower than the mean estimates obtained for previous generation Dexcom sensors, e.g., 9.7 min for Dexcom G4 PLATINUM and 7.7 min for Dexcom G4AP [37]. Our speculation is that such a reduction of the *τ* value is due to an improvement of both the sensing technology and the processing algorithm of the CGM device, which may compensate part of the delay produced by the plasma-interstitium kinetics.

Regarding the selection of the calibration error model, according to BIC the model (*poly*_2_, *poly*_0_) resulted in the optimal one to describe the calibration error for Dexcom G6. This model outperformed the exponential models, probably because it is more flexible than the exponential one, the latter describing either a monotonic increasing or a monotonic decreasing behavior. In previous studies, where the domain of validity of the model was limited to 12 h, the optimal calibration error models were (*poly*_1_, *poly*_1_) for Dexcom SEVEN Plus [36], (*poly*_1_, *poly*_0_) in day 1 and (*poly*_0_, *poly*_0_) in days 4 and 7 for Dexcom G4 and Dexcom G4AP [37], (*poly*_1_, *poly*_0_) for Dexcom G5 Mobile [22], and (*poly*_1_, *poly*_1_) for Medtronic Paradigm Veo Enlite [38]. In this study, a second-order calibration error model is selected, probably because more degrees of freedom are required to well describe the variability of the sensor sensitivity over a longer time horizon of 10 days, as in case of the Dexcom G6 sensor. 

Regarding the identification of the measurement noise model, the noise autocorrelation was best described by an autoregressive model of order 2, confirming the results of previous studies with sensors requiring in vivo calibrations [36,37,38]. 

Note that while a single optimal model was selected to describe the calibration error and the noise in all the sensors, a specific set of model parameters was estimated for each sensor to take into account the peculiarity of each sensor-patient behavior. The single-step identification procedure of model parameters implemented in this paper was shown to improve the parameter estimation compared to the two-step identification procedure used so far. In particular, the single-step identification procedure allows a statistically significantly better fit of the data (lower *RSS* values) than the two-step identification procedure, and improves the estimation of *τ* providing more physiologically sound estimates for this parameter. Indeed, the single-step identification procedure reduces the number of sensors with a *τ* value very close to 0, which is not reasonable from a physiologic point of view. Overall, the estimates of the parameters describing the calibration and the measurement noise errors are very similar between the single-step and the two-step identification procedures, confirming that the two-step identification approach used so far provides credible results.

## 5. Conclusions

In conclusion, in this paper we derived a new mathematical model describing the three main error components of a factory-calibrated CGM sensor (Dexcom G6), i.e., the distortion introduced by the BG-to-IG kinetics, the calibration error (e.g., caused by time-variability of sensor sensitivity), and the random measurement noise, and we improved the model parameter estimation by introducing the single-step identification procedure. A future development of the current study would be the validation of our results on other datasets acquired by the Dexcom G6. Our methodology can also be applied to datasets collected with other factory-calibrated sensors, like the Abbott FreeStyle Libre flash glucose monitoring system, caring about the selection of suitable calibration and noise models, which could be different from the optimal models selected in this study to describe the error of the Dexcom G6 sensor. Another interesting development would be to complement the error model developed in this study with a model of the sensor’s transient disconnections and compression artefacts [44]. The model identified in this paper can be integrated in T1D simulators, like the T1D patient decision simulator [22], as a tool to generate synthetic CGM data collected by factory-calibrated CGM sensors. The simulation models are becoming more and more advanced and reliable tools for the in silico testing of therapies based on medical devices [21]. Therefore, the equipment of T1D simulators with models of cutting-edge technologies, like the recently developed factory-calibrated CGM sensors, is a fundamental step to allow the use of these advanced tools to support the development of applications relying on such novel technologies.

## Figures and Tables

**Table 1:** Parameters’ estimates of the optimal model (i.e., *a*(*t*) = *poly*_2_, *b*(*t*) = *poly*_0_, *v*(*t*) = AR(2)) with the two-step and the single-step identification procedures. For each parameter we report the median and interquartile range (IQR) of the estimated values, as well as the percentage of sensors for which the CV of the estimation error is lower than 10% and 30%.

Parameter	Two-Step Identification			Single-Step Identification		
	Median [IQR]	CV < 10%	CV < 30%	Median [IQR]	CV < 10%	CV < 30%
τ [min]	3.10 [1.66–5.91]	86%	87%	3.78 [2.39–5.96]	85%	94%
a_0_ [unitless]	0.95 [0.87–1.03]	100%	100%	0.95 [0.86–1.03]	99%	99%
a_1_ [days^−1^]	0.002 [−0.034–0.027]	92%	99%	0.004 [−0.035–0.031]	41%	77%
a_2_ [days^−2^]	0.000 [−0.003–0.003]	94%	95%	0.000 [−0.003–0.003]	46%	78%
b_0_ [mg/dL]	7.30 [2.67–10.67]	90%	94%	6.35 [2.37–10.51]	54%	81%
α_1_ [unitless]	1.3 [1.17–1.37]	100%	100%	1.30 [1.15–1.37]	99%	99%
α_2_ [unitless]	−0.46 [−0.53–−0.33]	30%	90%	−0.42 [−0.53–−0.30]	95%	97%
σ [mg/dL]	3.20 [2.48–3.88]	-	-	3.19 [2.47–3.85]	-	-
